# Non-HLA Gene Polymorphisms in the Pathogenesis of Type 1 Diabetes: Phase and Endotype Specific Effects

**DOI:** 10.3389/fimmu.2022.909020

**Published:** 2022-06-21

**Authors:** Antti-Pekka Laine, Milla Valta, Jorma Toppari, Mikael Knip, Riitta Veijola, Jorma Ilonen, Johanna Lempainen

**Affiliations:** ^1^ Immunogenetics Laboratory, Institute of Biomedicine, University of Turku, Turku, Finland; ^2^ Institute of Biomedicine, Research Centre for Integrative Physiology and Pharmacology, and Centre for Population Health Research, University of Turku, Turku, Finland; ^3^ Department of Paediatrics, University of Turku and Turku University Hospital, Turku, Finland; ^4^ Pediatric Research Center, Children’s Hospital, University of Helsinki and Helsinki University Hospital, Helsinki, Finland; ^5^ Research Program for Clinical and Molecular Metabolism, Faculty of Medicine, University of Helsinki, Helsinki, Finland; ^6^ Tampere Center for Child Health Research, Tampere University Hospital, Tampere, Finland; ^7^ Department of Paediatrics, PEDEGO Research Unit, Medical Research Center, University of Oulu, Oulu, Finland; ^8^ Department of Children and Adolescents, Oulu University Hospital, Oulu, Finland; ^9^ Clinical Microbiology, Turku University Hospital, Turku, Finland

**Keywords:** type 1 diabetes, autoantibodies, seroconversion, follow-up-cohort, survival analysis

## Abstract

The non-HLA loci conferring susceptibility to type 1 diabetes determine approximately half of the genetic disease risk, and several of them have been shown to affect immune-cell or pancreatic β-cell functions. A number of these loci have shown associations with the appearance of autoantibodies or with progression from seroconversion to clinical type 1 diabetes. In the current study, we have re-analyzed 21 of our loci with prior association evidence using an expanded DIPP follow-up cohort of 976 autoantibody positive cases and 1,910 matched controls. Survival analysis using Cox regression was applied for time periods from birth to seroconversion and from seroconversion to type 1 diabetes. The appearance of autoantibodies was also analyzed in endotypes, which are defined by the first appearing autoantibody, either IAA or GADA. Analyzing the time period from birth to seroconversion, we were able to replicate our previous association findings at *PTPN22*, *INS*, and *NRP1.* Novel findings included associations with *ERBB3*, *UBASH3A*, *PTPN2*, and *FUT2*. In the time period from seroconversion to clinical type 1 diabetes, prior associations with *PTPN2*, *CD226*, and *PTPN22* were replicated, and a novel association with *STAT4* was observed. Analyzing the appearance of autoantibodies in endotypes, the *PTPN22* association was specific for IAA-first. In the progression phase, *STAT4* was specific for IAA-first and *ERBB3* to GADA-first. In conclusion, our results further the knowledge of the function of non-HLA risk polymorphisms in detailing endotype specificity and timing of disease development.

## Introduction

The HLA region on chromosome 6p21 is the major genetic risk factor for developing type 1 diabetes (T1D), and it confers around 50% of the overall genetic risk. The rest of the genetic risk is distributed among several loci throughout the genome. So far, at least 57 non-HLA T1D susceptibility regions have been confirmed (1), and the most recent study reported 78 genome-wide-significant regions (2). Many of the T1D risk associated genes affect immune cell or pancreatic β-cell functions (3).

Clinical T1D is preceded by a subclinical period during which β-cell destruction appears and impaired glucose tolerance emerges. The duration of this period varies strongly from weeks to years (4). The emergence of pre-diabetes and β-cell autoimmunity is marked by the emergence of the first islet-specific autoantibodies (AAB), of which those targeting insulin (IAA), glutamic acid decarboxylase 65 (GADA), and islet antigen-2 (IA-2A) are best characterized and can be used as markers of increased disease risk (5). IAA or GADA are most often found as the first appearing AAB and they at least partially seem to be associated with different mechanisms in the pathogenesis of T1D (known also as endotypes) (6). Advanced autoimmunity is indicated by the spreading of autoimmunity to cover at least two β-cell autoantigens. Many factors, including age, HLA class II genotypes, and certain non-HLA gene polymorphisms, affect the disease initiation, whereas other factors, including HLA class I genes, some non-HLA gene polymorphisms, and age at AAB seroconversion, are associated with enhanced progression to clinical T1D (3).

The Finnish Type 1 Diabetes Prediction and Prevention (DIPP) study is a birth cohort study, in which children are followed up from birth and monitored for the appearance of β-cell autoimmunity and the development of clinical T1D. We previously reported a survival analysis on 39 SNP markers from 37 T1D susceptibility loci in 521 persistently AAB-positive cases and 989 matched controls from the DIPP study (7). In this analysis, we set out to re-examine these prior findings by using the added power of the expanded cohort of 976 children with persistent AABs and 1910 matched controls. Altogether, 21 loci with prior evidence of association in the previous survival study (7) or in a family-based association study in the Finnish population (8) were chosen for analysis with the expanded cohort.

## Materials and Methods

### Subjects

All study subjects were participants in the Finnish Diabetes Prediction and Prevention Study (DIPP) (9), in which newborn children born in Turku, Oulu, and Tampere university hospitals are screened for HLA-conferred genetic risk for developing T1D. Both the original selection process, based on the typing of T1D-risk HLA-DQB1*03:02 and DQB1*02 and protection associated with DQB1*03:01 and DQB*06 02/3 alleles (10), and the development of the screening protocol during various phases of the study to higher sensitivity and specificity, including “full-house” HLA-DQB1 typing and selected HLA-DQA1 and HLA-DRB1 alleles (11), have been presented in detail before. Children with selected T1D-predisposing HLA genotypes are invited to a prospective follow-up study and subsequently screened for the appearance of AAB at regular clinic visits at 3–12-month intervals, depending on the age of the child and AAB positivity (10). In the original study protocol, children were first monitored for the appearance of islet cell antibodies (ICA) and, if testing positive, the biochemical AAB to insulin (IAA), glutamic acid decarboxylase 65 (GADA), and islet antigen‐2 (IA–2A) were analyzed from all available samples from such participants. Since 2003, all children have been tested for all four AAB at each clinical visit, as well as 1,006 children born between November 1994 and July 1997 (12). Progression to T1D was diagnosed according to the WHO criteria. The study was approved by the local ethics committee and written informed consent was obtained from the parents of the participating children.

The study cohort comprised 976 case subjects with β-cell autoimmunity, defined by having at least one persistently positive AAB (IAA, GADA, or IA–2A), and 1,910 AAB negative, clinically healthy control subjects. Controls were matched according to cases for sex, study center, and birth date (a range of two months before or after birth was included for matching) (**Table 1**). During the follow-up period, 560 cases developed positivity for multiple AABs and altogether 426 subjects progressed to T1D. The median age (interquartile range) was 10.47 (5.98–14.97) years in all cases. The median ages at seroconversion and for progression to clinical T1D (interquartile ranges) were 3.30 (1.49–5.11) years and 6.75 (3.68–9.82) years, respectively. The median follow-up time (interquartile range) for signs of autoimmunity and for developing clinical T1D was 10.83 (6.65–15.01) years in the controls. More details on the study subjects are presented in **Table S1**.

**Table 1:** Number of AAB positive cases and their matched controls in complete cohort **(A)** and endotype specific subgroups **(B)**.

A	No. of cases	% of cases	No. of controls	% of controls
All subjects	976	100	1,910	100
B				
IAA first AAB	330	33.8	643	33.7
GADA first AAB	329	33.7	644	33.7
Other^*^	236	24.2	463	24.2
Sum	895	91.7	1,750	91.6

^*^Multiple AABs or IA2A first.

### Genotyping

DNA for all study subjects was extracted from EDTA blood using salt extraction. For the new data set, the bulk of single-nucleotide polymorphisms (SNP) were genotyped at the Finnish Functional Genomics Centre core facility (Turku, Finland) using the Taqman OpenArray^®^ real-time PCR platform with QuantStudio 12K Flex Real-Time PCR System (Thermo Fisher Scientific, Waltham, Massachusetts, USA). Two SNPs (*CD226* rs763361, *NRP1* rs2666236) were genotyped using Taqman^®^ SNP genotyping Assays in QuantStudio™ 3 Real-Time PCR System (Thermo Fisher Scientific, Waltham, Massachusetts, USA). Only original Thermo Fisher reagents (assay and mastermix) were used in both Taqman SNP genotyping methods used. SNP genotyping for the previous data set of 521 cases and 989 controls, which were included in the current data set, was performed using the Sequenom (San Diego, California, USA) platform in the Genome Centre of Eastern Finland, University of Eastern Finland, Kuopio (7). Alleles were called using either QuantStudio™ Design and Analysis Desktop Software (QuantStudio 3 SNP analyses) or ThermoFisher Connect™ cloud Genotyping app (OpenArray SNP analyses).

There were no genotyping quality control issues after the subjects with subpar qPCR amplification were removed from the analysis. Genotyping success rate for all SNPs was high (>0.98) throughout the sample collection and none of the SNPs exhibited significant deviation from Hardy–Weinberg equilibrium (HWE) (p <0.05) in the control population.

The HLA DR-DQ haplotypes were determined using sequence-specific oligonucleotide probes as described earlier (13). Based on their HLA genotypes, the study participants were divided into four risk groups: strongly increased risk, moderately increased risk, slightly increased risk and a combined group for neutral or protective genotypes (13).

Allele frequencies for the SNPs under study were derived from the gnomAD v.2.1.1 European (Finnish) (14).

### Autoantibody Analysis

The protocols for determining ICA and biochemical AABs (IAA, GADA, and IA–2A) have been described earlier (15, 16).

### Statistical Analysis

Statistical analyses were conducted using the IBM SPSS Statistics 25.0 software package (Armonk, NY, USA). A Cox proportional hazards model was used to assess the effect of SNPs from birth to the emergence of β-cell autoimmunity and progression to T1D and from the time of seroconversion to clinical T1D. In addition to the main analysis with all case subjects, the same analyses were conducted separately in two subgroups of cases with either IAA or GADA as the first solely observed AAB at the seroconversion.

Both recessive and dominant inheritance models were tested, and the model showing the higher significance was selected for presentation (in **Table 2**) in each analysis category. Testing additive model was omitted since it does not show the effect size or direction for the tested genotype. The models were adjusted for HLA-conferred genetic risk by including the risk groups in the model. Resulting P-values were corrected for multiple testing by the number of SNPs being studied in each category using the Benjamini and Hochberg false discovery rate (FDR) step-up procedure (α = 0.05) (17) incorporated in the R package multtest (Katherine S. Pollard, Houston N. Gilbert, Yongchao Ge, Sandra Taylor, and Sandrine Dudoit. multtest: resampling-based multiple hypothesis testing, r package version 2.46.0.). A corrected P-value of <0.05 was considered statistically significant in the survival analysis. Genotyping success rate analysis for all study subjects and HWE calculations in the control population were performed using the PLINK 1.07 software package (URL: http://pngu.mgh.harvard.edu/purcell/plink/) (18).

**Table 2:** Cox regression survival analysis hazard ratios and corresponding *P*-values are shown for the time periods from birth to the appearance of the first AAB, from the first AAB to a clinical T1D diagnosis, and from birth to clinical T1D diagnosis.

Complete cohort				Birth to first AAB			First AAB to T1D			Birth to T1D		
A SNPs	Gene region	Major/minor allele	Risk allele	*P*-value	HR	95% CI	*P*-value	HR	95% CI	*P*-value	HR	95% CI
rs2476601	*PTPN22*	G/A	A	**2.2E−07**	1.43	1.25–1.63	**0.00020**	1.45	1.19–1.77	**4.4E−09**	1.80	1.48–2.20
rs7574865	*STAT4*	G/T	T	0.67	1.03	0.90–1.17	**0.0025**	1.35	1.11–1.63	0.16	1.15	0.95–1.39
rs2666236	*NRP1*	C/T	T	**0.0014**	1.24	1.09–1.42	0.65	1.05	0.85–1.29	0.049	1.23	1.00–1.51
rs689	*INS*	A/T	A	**7.9E−05**	1.32^*^	1.15–1.51	0.027	1.28^*^	1.03–1.59	**2.8E−05**	1.59^*^	1.28–1.98
rs2292239	*ERBB3*	C/A	A	**0.0057**	1.20	1.05–1.36	0.41	0.92	0.76 - 1.12	0.22	1.13	0.93–1.37
rs45450798	*PTPN2*	G/C	C	0.048	1.15	1.00–1.31	**0.00066**	2.41^*^	1.45–4.00	0.045	1.23	1.01–1.50
rs763361	*CD226*	C/T	T	0.26	1.09	0.94–1.25	**0.0021**	1.43	1.14–1.79	0.048	1.26	1.00–1.58
rs601338	*FUT2*	G/A	A	0.019	1.22^*^	1.03–1.44	0.026	1.26	1.03–1.54	**0.0088**	1.40^*^	1.09–1.80
rs9976767	*UBASH3A*	A/G	G	**0.0072**	1.20	1.05–1.37	0.37	1.10	0.90–1.34	**0.0036**	1.43^*^	1.12–1.82
**B** IAA first AAB												
rs2476601	*PTPN22*	G/A	A	**0.00077**	1.48	1.18–1.85	0.12	1.32	0.93–1.86	**0.0021**	2.47^*^	1.39–4.38
rs7574865	*STAT4*	G/T	T	0.96	1.01	0.80–1.26	**0.0012**	1.76	1.25–2.48	0.15	1.29	0.91–1.82
rs689	*INS*	A/T	A	0.017	1.34^*^	1.06–1.71	0.020	1.71^*^	1.09–2.69	**0.0013**	2.11^*^	1.34–3.31
rs2292239	*ERBB3*	C/A	A	0.06	1.24	0.99–1.54	0.025	0.67	0.47–0.95	0.88	1.03	0.73–1.45
**C** *GADA first AAB*												
rs2476601	*PTPN22*	G/A	A	0.31	1.13	0.89–1.45	0.76	0.92	0.54–1.57	0.69	1.12	0.65–1.91
rs7574865	*STAT4*	G/T	T	0.33	1.12	0.89–1.39	0.52	1.16	0.74–1.84	0.93	0.98	0.62–1.55
rs689	*INS*	A/T	A	0.50	0.84	0.50–1.41	0.43	1.59	0.50–5.07	0.45	0.64	0.20–2.04
rs2292239	*ERBB3*	C/A	A	0.08	1.22	0.97–1.52	**0.00068**	2.65^*^	1.51–4.66	0.014	2.01^*^	1.15–3.52

^*^Combined heterozygote and non-risk homozygote was used as reference genotype (recessive inheritance). Only results that remained significant after correction for multiple testing in the complete cohort are presented. Results for the complete cohort (A), for the cases with IAA as the first appearing AAB (B), and for the cases with GADA as the first appearing AAB (C) are shown separately. Significant *P*-values that survived correction for multiple testing are presented in bold and underlined. The non-risk homozygote was used as a reference genotype (dominant inheritance) in HR calculations for each SNP if not otherwise denoted in the HR-value.

## Results

All the SNPs being studied (**Table 3**) were established risk markers of T1D. The more particular aim of this follow-up study was to determine whether some markers were specifically associated with the early phase of autoimmunity development or its progression to a clinical diagnosis of T1D and whether they were specific for endotypes characterized by either IAA or GADA as the first autoantibody to appear.

**Table 3:** The genetic location and allele frequencies of the studied SNPs.

SNP	Gene	Chromosome band	Position	Minor/Major allele	Risk allele	Risk allele frequency^*^
rs630115	*LOC646538*	1p31.1	81100482	A/G	G	0.672
rs2476601	*PTPN22*	1p13.2	113834946	A/G	A	0.149
rs2816316	*RGS1*	1q31.2	192567683	G/T	T	0.856
rs3087243	*CTLA4*	2q33.2	203874196	A/G	G	0.667
rs7574865	*STAT4*	2q32.2	191099907	T/G	T	0.217
rs1990760	*IFIH1*	2q24.2	162267541	C/T	T	0.572
rs17388568	*ADAD1*	4q27	122408207	A/G	A	0.409
rs3757247	*BACH2*	6q15	90247744	A/G	A	0.365
rs6920220	*TNFAIP3*	6q23.3	137685367	A/G	A	0.187
rs12722495	*IL2RA*	10p15.1	6055320	G/A	A	0.941
rs2104286	*IL2RA*	10p15.1	6057082	G/A	A	0.810
rs2666236	*NRP1*	10p11.2	33129944	T/C	T	0.376
rs689	*INS*	11p15.5	2160994	T/A	A	0.797
rs3184504	*SH2B3*	12q24.1	111446804	T/C	T	0.397
rs2292239	*ERBB3*	12q13.2	56088396	A/C	A	0.305
rs3825932	*CTSH*	15q25.1	78943104	T/C	C	0.614
rs12708716	*CLEC16A*	16p13.1	11086016	G/A	A	0.643
rs763361	*CD226*	18q22.2	69864406	T/C	T	0.444
rs45450798	*PTPN2*	18p11.2	69864406	C/G	C	0.147
rs601338	*FUT2*	19q13.3	48703417	A/G	A	0.374
rs9976767	*UBASH3A*	21q22.3	42416281	G/A	G	0.335

^*^Allele frequencies derived from the gnomAD v2.1.1 European (Finnish) GRCh37.

**Table 2** contains results for the loci that have shown a significant association at least in one tested time period within either the full data set (A) or the IAA-first (B) or GADA-first (C) subsets. Complete results for all tested loci are shown in **Table S2**. Survival analysis of the entire follow-up period from birth to clinical T1D using multivariate Cox regression demonstrated significant associations with *PTPN22, INS, FUT2*, and *UBASH3A* (p = 4.4E−9, 2.8E−5, 0.0088, and 0.0036, respectively) (**Table 2A**). The known risk allele was associated with increased disease development in all loci.

### Effects of Non-HLA Gene Polymorphisms in Different Phases of Autoimmunity Development

*PTPN22*, *INS*, and *UBASH3A* also demonstrated a significant association (after correction for multiple testing) with the appearance of the first islet-specific AAB when followed from birth (p = 2.2E−07, 7.9E−05, and 0.0072, respectively), but *FUT2* was not significant after correction for multiple testing (p = 0.019). Additionally, *ERBB3* and *NRP1*, which did not show an association in the analysis from birth to T1D, presented significant associations with the appearance of the first islet-specific AAB (p = 0.0057 and 0.0014, respectively).

When the further period from seroconversion to clinical T1D was analyzed, four loci presented significant associations (*PTPN22, PTPN2, STAT4*, and *CD226*, p = 0.00020, 0.00066, 0.0025, and 0.0021, respectively). In all these loci, the known risk allele increased progression to diabetes. Of these four loci, *PTPN22* was the only one detected in the analysis of the whole period from birth to T1D.

### The Associations of Non-HLA SNPs With Disease Pathogenesis in Relation to Disease Endotypes

To explore the genetic heterogeneity of disease endotypes, the cases and respective controls were analyzed separately based on the initial appearing AAB (IAA or GADA). When the whole follow-up period from birth to clinical T1D was studied, significant associations were observed with *PTPN22* and *INS* in the IAA-first group (p = 0.0021 and 0.0013, respectively) (**Table 2B**).

When the follow-up period from birth to AAB seroconversion was analyzed, *PTPN22* was the only locus significantly associated with the IAA-first group (p = 0.00077). In the progression from AAB positivity to clinical T1D in the IAA subgroup, a significant association of *STAT4* with disease risk was observed (p = 0.0012).

When follow-up periods from birth to clinical T1D or from birth to AAB seroconversion were studied in the subgroup with GADA as the first AAB, no significant association was detected. In the progression from AAB positivity to clinical diabetes, *ERBB3* showed a significant association in this subgroup (p = 0.00068) (**Table 2C**).

An interesting, albeit non-significant observation, was the opposite direction of *ERBB3* and *FUT2* effects in the progression phase of the IAA-first vs. GADA-first subgroups. The risk alleles of both loci showed association with a protective effect in the IAA-first subgroup (HR = 0.67, HR = 0.60, respectively), and risk effect in the GADA-first subgroup (HR = 2.65, HR = 1.89, respectively), but these observations were not significant after correction for multiple testing except for the *ERBB3* GADA-first association (p = 0.00068) (Table S2).

## Discussion

The number of Finnish DIPP follow-up study subjects with persisting autoimmunity analyzed here has almost doubled since our previous report on non-HLA loci affecting initiation and development of T1D-specific autoimmunity (7). The increased statistical power of the expanded data set allowed new findings on the effect of non-HLA gene polymorphisms in various phases of the autoimmune process and in different endotypes of the autoimmune process leading to clinical T1D.

As all gene polymorphisms being studied here are confirmed T1D risk genes, it is conceivable that they affect survival from birth to clinical T1D. Therefore, the lack of such evidence in this analysis could plausibly demonstrate insufficient power to detect small effects, and in fact, when the follow-up period from birth to clinical T1D was examined, most of the polymorphisms under study did not show an association. The effect locus conveys on disease susceptibility can be restricted to either the initiation of autoimmunity or the spreading of autoimmunity, which in part may decrease the power to detect significant association signals in the entire follow-up period. For that reason, the main objective of the survival analysis in the whole cohort was to examine whether the genes studied exert their effects in the phase of islet autoimmunity initiation or in the progression from AAB positivity to clinical disease. We defined the progression period from the first AAB seroconversion to clinical diabetes to involve both the spreading of autoimmunity and the β-cell destruction, and therefore the risk effect has the potential to be mediated by different mechanisms.

The analysis of the time period from birth to the appearance of the first AAB replicated associations in our earlier study (7) with *PTPN22* and *INS*. The prior suggestive association (that did not survive the correction for multiple testing) observed for *NRP1*, was now confirmed as a significant association. On the other hand, the prior significant association evidence found with *IFIH1* was now reduced to a suggestive association (**Table S2**). Novel findings in the current study were significant associations between the appearance of AABs and *ERBB3, NRP1*, and *UBASH3A*. Three of these loci have shown an association also in other studies: *PTPN22* and *INS* in TEDDY (19), and *PTPN22* and *UBASH3A* in DAISY (20). *IFIH1* has been reported to be associated with progression from AAB positivity (20, 21), but we did not observe such an association in our cohort.

We have found association evidence earlier for *IL2RA*, *CD226, PTPN2, LOC646538*, and *PTPN22* in the analysis of the time period from AAB positivity to clinical T1D (7, 22). The previous significant association with *PTPN2* was replicated in this study and earlier suggestive evidence with *PTPN22* and *CD226* was now confirmed with significant associations. A significant association with progression from seroconversion to clinical T1D and *STAT4* was a novel observation.

The discovery of specific gene effects in separate analyses of endotypes, characterized by autoimmunity initiated with either IAA or GADA as the first AAB, is expected to produce important information on the pathogenetic mechanisms (6). Novel, endotype-specific significant associations were also found in this study. As in the whole cohort, *PTPN22* demonstrated the strongest association with the development of autoimmunity in the subgroup, with IAA as the first AAB. However, in the GADA-first group, no significant effect was seen, unlike in the TEDDY study (23). As was the case in our earlier study and the TEDDY study, *INS* was associated with the IAA-first endotype (7, 22). In the combined cohort for AAB appearance, we could not replicate the *SH2B3* association observed in TEDDY (24). The other known T1D risk gene, *TNFAIP3*, which in TEDDY was associated with progression to clinical disease, (25) did not show any association in the current study.

The progression from AAB positivity to clinical T1D is connected with the decreasing ability of β-cells to produce insulin, and its rate is associated with alleles in class I HLA genes (26, 27) and with several genes outside the HLA region. A significantly steeper decrease in the first-phase insulin response was observed during follow-up in AAB-positive DIPP children in an earlier study with risk genotypes of *CTSH, PTPN2, IKZF4*, and *FUT2* genes (28). *PTPN2* and *ERBB3* (which is in tight linkage disequilibrium with *IKZF4*) showed a significant association with progression from seroconversion to clinical disease in the current study, but the associations with *ERBB3* (**Table 2A**) and *FUT2* were seen only in the GADA-first group, and the *FUT2* association was only suggestive (**Table S2**).

With a few exceptions, the mechanistic effects of T1D risk genes are largely unknown (3). *PTPN22* encodes protein tyrosine phosphatase, which acts as a negative regulator of T-cell receptor signaling. Its variant rs2476601, which causes a missense R620W mutation, increases the risk of multiple autoimmune diseases and is associated with a multitude of cell population-specific effects, some of which are contradictory. For example, increased T-effector activation has been reported (29), but also a compensatory increased frequency of naive regulatory T cells, important, in suppression of autoimmunity (30, 31). This polymorphism also modulates multiple other pathways, including B-cell and Toll-like receptors, NLRP3, and integrin signaling (3). It therefore seems likely that these multiple effects may affect both the initiation and progression phases of the disease. Our data also support a model where rs2476601 has multiple effects in different cell populations throughout disease pathogenesis.

In the current data, *STAT4* was significantly associated with the progression from seroconversion to clinical T1D, specifically among IAA-first subjects. This gene is involved in signal transduction and activates transcription in response to proinflammatory cytokines (32, 33). Additionally, it participates in T helper 1 cell regulation and is expressed in activated monocytes, dendritic cells, and macrophages, and its inhibition in the NOD mouse model prevents autoimmune diabetes (32). A meta-analysis of the significance of *STAT4* rs7574865 T1D risk allele T has confirmed its association with T1D in multiple populations, in addition to the already earlier found association with other autoimmune diseases (34). A recent study also reported a higher STAT4 expression level in peripheral blood mononuclear cells in T1D cases compared to controls and an association of the rs7574865 T allele with younger age at the T1D diagnosis (35). The finding of a young age at diagnosis in risk allele positive children concurs with our finding of rs7574865 risk allele T increasing development of AABs and with the association of IAA as the first AAB because IAA dominates over GADA as the first AAB among the youngest seroconverted children, which is also reflected in a younger age at diagnosis (36).

Strong associations of *NRP1* and *UBASH3A* with AAB induction were seen for the first time in the DIPP cohort in this study (7). The *NRP1* gene encodes neuropilin 1, which is a co-receptor for vascular endothelial growth factor (VEGF) and is essential for its binding and activity in endothelial cells, including pancreatic islets as well. Various isoforms have been implicated in affecting β-cell regeneration (37). The effects of *NRP1* polymorphism on the large spectrum of VEGF functions are, however, still largely unknown. T1D associated SNP alleles in *UBASH3A* increase its expression in primary human CD4+ T cells and inhibit NF-κB signaling, resulting in reduced IL-2 production, which might increase the probability of initiating autoimmunity (38).

The *ERBB3* risk allele is associated with the appearance of AABs in the whole cohort in our study. The hazard ratio (HR) for the whole cohort is very similar to the HR observed in both the IAA-first and GADA-first subgroups, although it does not quite reach significance in smaller subgroups. However, when the period from seroconversion to clinical T1D is observed, the *ERBB3* risk allele A shows effects in opposite directions between the IAA-first and GADA-first subgroups, with a significant risk effect in the GADA-first group and a protective suggestive effect (non-corrected p <0.05) in the IAA-first subgroup. The whole cohort does not show an association with progression to T1D, probably because the contradictory effects in the two subgroups neutralize each other. It thus seems possible that *ERBB3* rs2292239 or the region around it is associated with at least two different effector mechanisms. The 12q13 region includes a group of genes with very strong linkage disequilibrium and, e.g., *IKZF4* and *ERBB3* risk alleles both have a very similar effect on T1D susceptibility (8, 39), but independent SNP associations have also been detected in this region (39). A gene expression study of six genes in the region demonstrated a higher expression of ERBB3 on antigen-presenting cells after LPS, poly C, or CpG stimulation. The study also observed a higher proportion of ERBB3 positive monocytes and dendritic cells, and the proportion of these cells correlated with the ability of antigen-presenting cells to induce T-cell proliferation (40). However, the exact mechanism of how these differences would affect the risk of T1D remains unclear. Interestingly, a recent paper from the Belgian Diabetes Registry described that *ERBB3/IKZF4* risk alleles increased the progression from single to multiple AABs, but this was seen only in female relatives (41). Alleged multiple independent effects emphasize the possibility of the importance of the *IKZF4* gene, which encodes the Eos molecule, which in turn in mouse models seems to be important for T-reg cell functions and suppression of autoimmunity (42, 43).

*CD226* was significantly associated with the progression from seroconversion to clinical T1D. Neither IAA- nor GADA-first subjects showed a significant association, suggesting that the result of the entire cohort contributes to both IAA and GADA-initiated disease pathways. The HR effect size was similar in both groups to that observed in the whole cohort. CD226 is a co-stimulatory molecule known to augment effector/memory T cell and NK cell activation, and the disease-associated variant may promote the MARK/ERK pathway and thus enhance the production of inflammatory and cytotoxic molecules (3). It might therefore play a major role in the phase of β-cell destruction by immune cells. Similar to *CD226*, also *PTPN2* was strongly associated with progression from seroconversion to clinical T1D, but not with AAB induction or with IAA or GADA associated disease. This is in accordance with its role in sensitization of pancreatic β-cells to interferon-induced apoptosis (44).

We were able to validate most of our earlier observations in this expanded follow-up study and found new associations in some genes, suggesting that they affect either specific stages of the development of T1D and/or one of the two major endotypes characterized by either IAA or GADA as the first AAB. Many of these findings are in agreement with reports from other follow-up studies, whereas others await confirmation. Differences between participating populations and study protocols may explain differences in identified associations, e.g., between the international TEDDY study and the Finnish DIPP study. Children eligible for the TEDDY study from the general population represented a few high and moderate risk HLA genotypes, covering together around 42% of Finnish children with T1D, whereas the DIPP study recruited a larger follow-up group, covering 60–70% of the genotypes found in T1D children, including also lower HLA risk genotypes. A higher frequency of non-HLA gene risk genotypes like *INS* has actually been found in children with T1D, whose HLA genotypes are associated with a relatively low T1D risk (45, 46). There are also differences in the definitions of various stages of autoimmunity between studies. We analyzed the progression from the appearance of the first AAB to the diagnosis of clinical diabetes, whereas the TEDDY study analyzed the progression from the appearance of multiple AABs to the diagnosis, which is a shorter period (25).

In addition to sample size restrictions, there are other limitations to the study. The progress to autoantibody positivity and to the clinical diagnosis has been found to be associated with high genetic risk scores combining HLA and non-HLA markers (47) and it might be useful in the future to somehow adjust for the individual genetic risk. However, the recruitment criterion for the DIPP study is defined by the HLA class II genotype associated with high or moderate T1D risk. The appearance of islet autoantibodies, which is a prerequisite for the development of the disease, is mainly associated with high-risk class II HLA genotypes (48). In conclusion, our results contribute to a more specific analysis of the function of non-HLA T1D risk polymorphisms by detailing disease endotype specificity and timing of the disease process. The numbers of participants in follow-up cohorts who have developed AABs and especially T1D, are limited and one can expect only the strongest associations to turn out to be significant although the numbers of AAB-positive subjects and diagnosed T1D cases, are as far as we know, higher than ever published earlier in follow-up studies of this type. A deeper understanding of the mechanisms through which risk effects are mediated will increase our understanding of the development of T1D and potentially open new avenues to better treatments and even disease prevention.

## Data Availability Statement

The datasets presented in this article are not readily available because of data privacy regulations. Requests to access the datasets should be directed to DIPP Steering Committee, johanna.lempainen@utu.fi.

## Ethics Statement

The studies involving human participants were reviewed and approved by The Ethics Committees at the three participating University Hospitals, Turku, Oulu, and Tampere. Written informed consent to participate in this study was provided by the participants’ legal guardian/next of kin. Written informed consent was obtained from the individual(s), and minor(s)’ legal guardian/next of kin, for the publication of any potentially identifiable images or data included in this article.

## Author Contributions

A-PL, MV, JI, and JL designed the experiments. The manuscript was written by A-PL, MV, JI, and JL. A-PL, MV, JI, JT, MK, JL, and RV contributed in reviewing and editing the article. A-PL, MV, JI, and JL designed the study and drafted the manuscript. A-PL and MV performed the experiments and analyzed the data. A-PL, JI, JT, MK, and RV acquired the data and critically reviewed the manuscript. A-PL is the guarantor of this work and, as such, had full access to all the data in the study and takes responsibility for the integrity of the data and the accuracy of the data analysis. All authors listed have made a substantial, direct, and intellectual contribution to the work and approved it for publication.

## Funding

This work was funded by the Sigrid Jusélius Foundation, the Finnish Medical Foundation, the Juvenile Diabetes Research Foundation (JDRF), grant number 3-SRA-2020-955-S-B and the Academy of Finland grant number 286765.

## Conflict of Interest

The authors declare that the research was conducted in the absence of any commercial or financial relationships that could be construed as a potential conflict of interest.

## Publisher’s Note

All claims expressed in this article are solely those of the authors and do not necessarily represent those of their affiliated organizations, or those of the publisher, the editors and the reviewers. Any product that may be evaluated in this article, or claim that may be made by its manufacturer, is not guaranteed or endorsed by the publisher.
